# Two-dimensional ultrasound measurements vs. magnetic resonance imaging-derived ventricular volume of preterm infants with germinal matrix intraventricular haemorrhage

**DOI:** 10.1007/s00247-019-04542-x

**Published:** 2019-11-06

**Authors:** Casper Beijst, Jeroen Dudink, Rens Wientjes, Isabel Benavente-Fernandez, Floris Groenendaal, Margaretha J. Brouwer, Ivana Išgum, Hugo W. A. M. de Jong, Linda S. de Vries

**Affiliations:** 1 Department of Neonatology, Wilhelmina Children’s Hospital/University Medical Center Utrecht, Utrecht University, Utrecht, The Netherlands; 2 Department of Medical Technology and Clinical Physics, University Medical Center Utrecht, Utrecht University, Utrecht, The Netherlands; 3 Department of Radiology and Nuclear Medicine, University Medical Center Utrecht, Utrecht University, P.O. Box 85500, 3508 GA Utrecht, The Netherlands; 4 Brain Center Rudolf Magnus, University Medical Center Utrecht, Utrecht University, Utrecht, the Netherlands; 5 Neonatology Department, “Puerta del Mar” University Hospital, Cadiz, Spain; 6 Image Sciences Institute, University Medical Center Utrecht, Utrecht University, Utrecht, The Netherlands

**Keywords:** Brain, Germinal matrix intraventricular haemorrhage, Infants, Magnetic resonance imaging, Ultrasound, Ventricular volume

## Abstract

**Background:**

Post-haemorrhagic ventricular dilatation can be measured accurately by MRI. However, two-dimensional (2-D) cranial US can be used at the bedside on a daily basis.

**Objective:**

To assess whether the ventricular volume can be determined accurately using US.

**Materials and methods:**

We included 31 preterm infants with germinal matrix intraventricular haemorrhage. Two-dimensional cranial US images were acquired and the ventricular index, anterior horn width and thalamo-occipital distance were measured. In addition, cranial MRI was performed. The ventricular volume on MRI was determined using a previously validated automatic segmentation algorithm. We obtained the correlation and created a linear model between MRI-derived ventricular volume and 2-D cranial US measurements.

**Results:**

The ventricular index, anterior horn width and thalamo-occipital distance as measured on 2-D cranial US were significantly associated with the volume of the ventricles as determined with MRI. A general linear model fitted the data best: ∛ventricular volume (ml) = 1.096 + 0.094 × anterior horn width (mm) + 0.020 × thalamo-occipital distance (mm) with R^2^ = 0.831.

**Conclusion:**

The volume of the lateral ventricles of infants with germinal matrix intraventricular haemorrhage can be estimated using 2-D cranial US images by application of a model.

## Introduction

Very-low-birth-weight (<1,500 g) infants are at increased risk of developing a germinal matrix intraventricular haemorrhage, which occurs in 20–25% of cases [1, 2]. Approximately 35% of the infants who have a germinal matrix intraventricular haemorrhage develop post-haemorrhagic ventricular dilatation [3]. In severe cases of post-haemorrhagic ventricular dilatation, there is an increased risk of raised intracranial pressure and subsequent brain damage, and an intervention such as a lumbar puncture, the placement of a subcutaneous reservoir or a ventriculo-peritoneal shunt might be required [4, 5].

To diagnose and monitor post-haemorrhagic ventricular dilatation and to assess whether intervention is required, two-dimensional (2-D) cranial US is used. The most important measurements are the ventricular index, anterior horn width and thalamo-occipital distance [6]. These measurements aid in managing post-haemorrhagic ventricular dilatation, i.e. in determining when to perform lumbar punctures, whether to insert a ventricular reservoir, how often and how much cerebrospinal fluid to withdraw and whether to insert a ventriculo-peritoneal shunt. Another important prognostic imaging marker is the ventricular volume. This, however, cannot be measured using 2-D cranial US. Magnetic resonance imaging (MRI) is generally considered to be the most accurate way to determine the ventricular volume in neonates. Not all premature babies undergo MR scanning. When they do, it is usually performed once or twice during the neonatal period and at most centers the MRI is performed around term-equivalent age.

Although MRI provides high-resolution and anatomical detail, MRI has limitations. It can be challenging to maintain physiological stability during an MRI examination. Moreover, there are potential risks for infants during transfer to the MR unit, sedation might be required, and the examination is time-consuming and often costly. Also, MRI cannot be easily repeated for monitoring and follow-up. Two-dimensional cranial US is routinely used on the neonatal intensive care unit to diagnose and assess the progression of post-haemorrhagic ventricular dilatation. Therefore, it would be advantageous if US could also be used for ventricular volume assessment [7].

The purpose of the study was to assess whether the ventricular volume can be determined accurately using US, to pave the way for a US-derived measurement of ventricular volume. The rationale is that the ventricular volume might be more directly linked to intracranial pressure, which in turn is considered to be the actual cause of additional damage to the surrounding white matter.

## Materials and methods

### Study population

For this study, we included preterm infants with a germinal matrix intraventricular haemorrhage and a gestational age less than 31 weeks who were admitted to the Level 3 neonatal intensive care unit between 2007 and 2013 and had an MRI examination at term-equivalent age along with coronal and sagittal 2-D cranial US examination within 3 days of the MRI. Because we used clinically obtained anonymized data, our medical ethics committee waived the requirement for written informed parental consent for participation in the study.

### Ultrasonography

The infants routinely underwent cranial US for the assessment of ventricular dilatation. Two-dimensional cranial US imaging was performed by neonatologists and physician assistants with experience varying between 1 year and 25 years. The US images were reviewed by neonatologists with 10 years to 25 years of experience in performing cranial ultrasound. For comparison of 2-D cranial US and MR images, we included only the US images that were acquired within 3 days of the MRI examination. Ultrasound images were acquired using a Xario XG, Aplio MX or Aplio ultrasound machine (Toshiba Medical Systems, Tokyo, Japan) with PST65-AT (9S4) transducer operating in a frequency range between 4.2 MHz and 9 MHz, or an ATL-5000 ultrasound machine (Philips Medical Systems, Best, The Netherlands) with a C8–5 transducer operating in a frequency range between 5 MHz and 8 MHz.

In general, US imaging was performed within 6 h of admission, at least three times in the first week after birth, then weekly till discharge to a Level 2 hospital, and again at term-equivalent age. Criteria for post-haemorrhagic ventricular dilatation were a ventricular index >97th percentile according to Levene and Starte [8], anterior horn width >6 mm or thalamo-occipital distance >24 mm, according to Davies et al. [9].

**Fig. 1:**
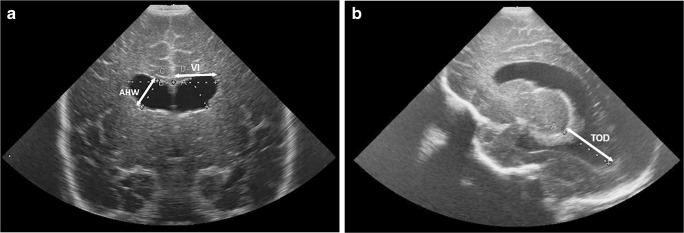
Imaging measurements in a boy who was born at 28 weeks of gestational age and developed post-haemorrhagic ventricular dilatation. He was examined at a postnatal age of 12 weeks. **a**, **b** Coronal (**a**) and sagittal (**b**) US images show how the ventricular index (*VI*), anterior horn width (*AHW*) and thalamo-occipital distance (*TOD*) were determined for this study

For the assessment of ventricular volume, we acquired three linear measurements from the cranial US images: the ventricular index (defined as the distance between the falx and the lateral wall of the anterior horn in the coronal plane) [8], the anterior horn width (defined as the diagonal width of the anterior horn measured at its widest point in the coronal plane) [9], and the thalamo-occipital distance (defined as the distance between the outermost point of the thalamus at its junction with the choroid plexus and the outermost part of the occipital horn in the parasagittal plane) [6], as shown in Fig. 1.

The ventricular index, anterior horn width and thalamo-occipital distance were measured by multiple observers. When measurements of a single infant were performed by more than one observer, we used measurements of the most experienced observer for further analysis. We calculated the intraclass correlation coefficient for the images where multiple observer measurements were available.

### Magnetic resonance imaging

MR images were acquired at term-equivalent age on an Achieva 3.0-tesla (T) MR system (Philips Healthcare, Best, The Netherlands). At our institution, all preterm infants with a germinal matrix intraventricular haemorrhage with a gestational age less than 28 weeks undergo MRI. In those with a gestational age >28 weeks, MRI is performed when a large intraventricular haemorrhage is diagnosed with US. In infants with US abnormalities, such as a large intraventricular haemorrhage, MRI is also performed to obtain more information on additional lesions in the white matter and the cerebellum. Different exam protocols were used for infants scanned before and after May 2008. Before May 2008, infants were scanned with an axial 3-D T1-weighted gradient echo sequence using a repetition time of 9.4 ms, echo time of 4.6 ms, a slice thickness of 2.0 mm without gap; and an axial T2-weighted spin-echo sequence with a repetition time of 6,293 ms, echo time of 120 ms and slice thickness of 2.0 mm. As of June 2008, T1-weighted images were acquired using a coronal 3-D gradient echo scan with a repetition time of 9.5 ms, echo time of 4.6 ms, slice thickness of 1.2 mm and no gap; and T2-weighted images were acquired using a coronal spin-echo scan with a repetition time of 4,847 ms, echo time of 150 ms, slice thickness of 1.2 mm and no gap. More details on the MRI protocol have been published [10].

**Fig. 2:**
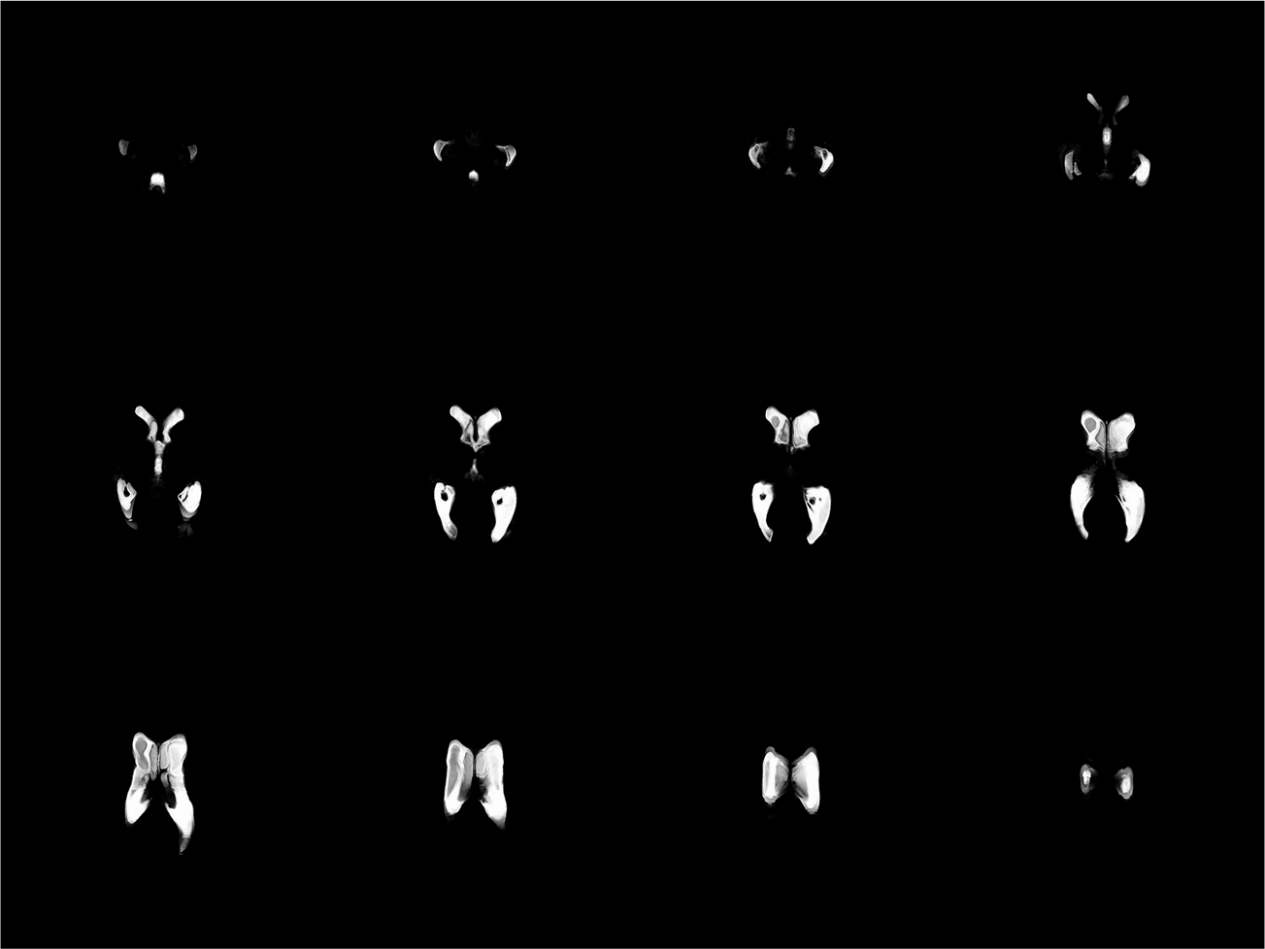
MRI segmentation in a boy born at 28 weeks of gestation who developed post-haemorrhagic ventricular dilatation and was examined at a postnatal age of 12 weeks. The montage of images shows the MRI segmentation of the ventricles in several transaxial slices

We used a probabilistic brain segmentation algorithm as described by Anbeek et al. [11] to perform the automatic segmentation of the brain volume. The algorithm not only segments the ventricles, but also separately classifies the white matter, cortical and deep grey matter, brainstem, cerebellum and extracerebral cerebrospinal fluid. The algorithm does this by a supervised voxel classification considering the intensity of T1- and T2-weighted images as well as the location of the voxel within the brain. The resulting segmentations are obtained by summation of the individual voxels with sufficient calculated probability of belonging to a certain tissue type. A study was previously performed to validate the resulting segmentations against human observers, and found no significant differences in the segmented volumes in five preterm infants [12]. The volume of the automatically segmented ventricles (lateral, 3rd and 4th ventricles) was obtained by summation of the number of voxels weighted by their respective probabilities as obtained from the classification algorithm. Because the training of automatic segmentation algorithms for pathological anatomy remains a challenge, we reviewed all automatic segmentations and manually corrected wherever necessary. An MRI segmentation of the ventricles is shown in Fig. 2.

### Data analysis

For data analysis we used the SPSS statistical software package version 21 (IBM, Armonk, NY). We performed linear regression analysis to study the association between the ventricular volume and the US measurements. The ventricular volume on MRI was determined for the left and right separately, and compared to the ventricular index, anterior horn width and thalamo-occipital distance measurements of that respective side of the brain. Furthermore, we fitted a general linear model with multiple covariates using forward selection with a *P*-value <0.05. We performed a log transformation and a transformation with the cube root (∛) of the ventricular volume to improve homoscedasticity (the homogeneity of the variance) and obtain a normal distribution of the residuals. We performed the linear US measurements, the volume measurements on MRI, and the model fitting for both the left and right side separately. In this way, we could optimally incorporate the measurements of infants with asymmetrical ventricles in our model.

### Model validation

For validation purposes we compared our model to the model of Benavente-Fernandez et al. [13]. This model predicts the ventricular volume as measured by 3-D cranial US, using linear 2-D cranial US measurements in infants with post-haemorrhagic ventricular dilatation.

## Results

In total, 31 preterm infants with germinal matrix intraventricular haemorrhage were eligible for inclusion in this study, and 14 infants (45%) developed post-haemorrhagic ventricular dilatation (Table 1). T1- and T2-weighted MR images were obtained at term-equivalent age (median 41.1 weeks, range 40.3–43.1 weeks). Ventricular volumes were obtained by automatic segmentation. The automatic segmentation was manually reviewed and corrected where necessary.

**Table 1:** Patient characteristics

		Mean	Standard Deviation	Minimum	Maximum	Count
Gestational age (weeks)		27.2	1.5	24.9	30.4	
Birth weight (g)		1,039	288	670	1,775	
Post-menstrual age MRI (weeks)		41.3	0.8	40.3	43.1	
Weight at term-equivalent age (g)		3,496	480	2,285	4,735	
Grade intra-ventricular haemorrhage	1					0
	2					25
	3					6
Ventriculo-peritoneal drain	Yes					2
	No					29
Gender	Female					11
	Male					20

Within 3 days of the MRI examination, coronal and sagittal cerebral cranial US images were acquired (Fig. 1); at this time no ventricular taps from a Rickham reservoir were performed. For each child the ventricular index, anterior horn width and thalamo-occipital distance were measured on both sides and used for further statistical analysis as separate measurements (Table 2). We determined the intraclass correlation coefficient (ICC) using the one-way random effects models, yielding ICCs of 0.924 (95% confidence interval [CI] 0.854–0.961), 0.988 (95% CI 0.975–0.994) and 0.984 (95% CI 0.951–0.995) for the ventricular index, anterior horn width and thalamo-occipital distance, respectively.

**Table 2:** MRI and two-dimensional cranial US measurements at term-equivalent age (*n*=31)

	Mean	Median	Minimum	Maximum	SD
Ventricular volume, MRI (ml)	8.1	6.1	2.0	30.3	5.9
Ventricular index, cranial US (mm)	13.6	13.7	8.4	19.5	2.0
Anterior horn width, cranial US (mm)	4.4	3.7	0.6	12.1	2.6
Thalamo-occipital distance, cranial US (mm)	20.7	20.4	7.4	46.0	8.0

*SD* standard deviation

**Fig. 3:**
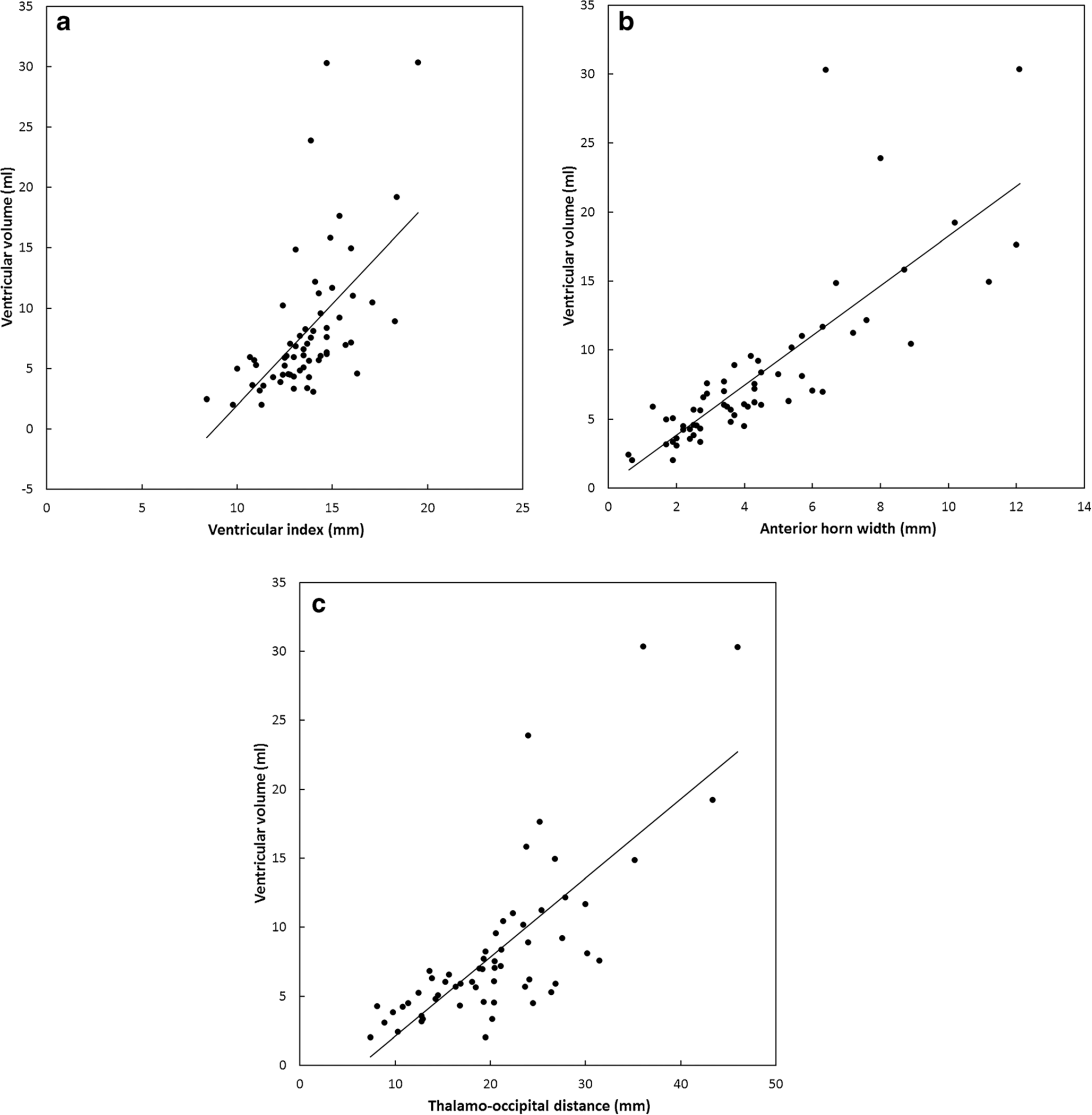
Magnetic resonance imaging (MRI)-derived ventricular volume. **a**–**c** Plotted as a function of ventricular index (**a**), anterior horn width (**b**) and thalamo-occipital distance (**c**). We performed a linear fit and the resulting functions were: ventricular volume (ml) = −16.1+1.77 × ventricular index (mm) (R^2^ = 0.334); ventricular volume (ml) = −0.01+1.84 × anterior horn width (mm) (R^2^ = 0.658); and ventricular volume (ml) = −3.70+0.576 × thalamo-occipital distance (mm) (R^2^ = 0.574)

Figure 3 shows the ventricular volume plotted as a function of ventricular index, anterior horn width and thalamo-occipital distance. We performed a linear fit to add a trendline to the plot, which resulted in the following relations for each of the covariates: ventricular volume (ml) = −16.1+1.77 × ventricular index (mm) (R^2^ = 0.334); ventricular volume (mm) = −0.01+1.84 × anterior horn width (mm) (R^2^ = 0.658); and ventricular volume (ml) = −3.70 + 0.576 × thalamo-occipital distance (mm) (R^2^ = 0.574).

**Fig. 4:**
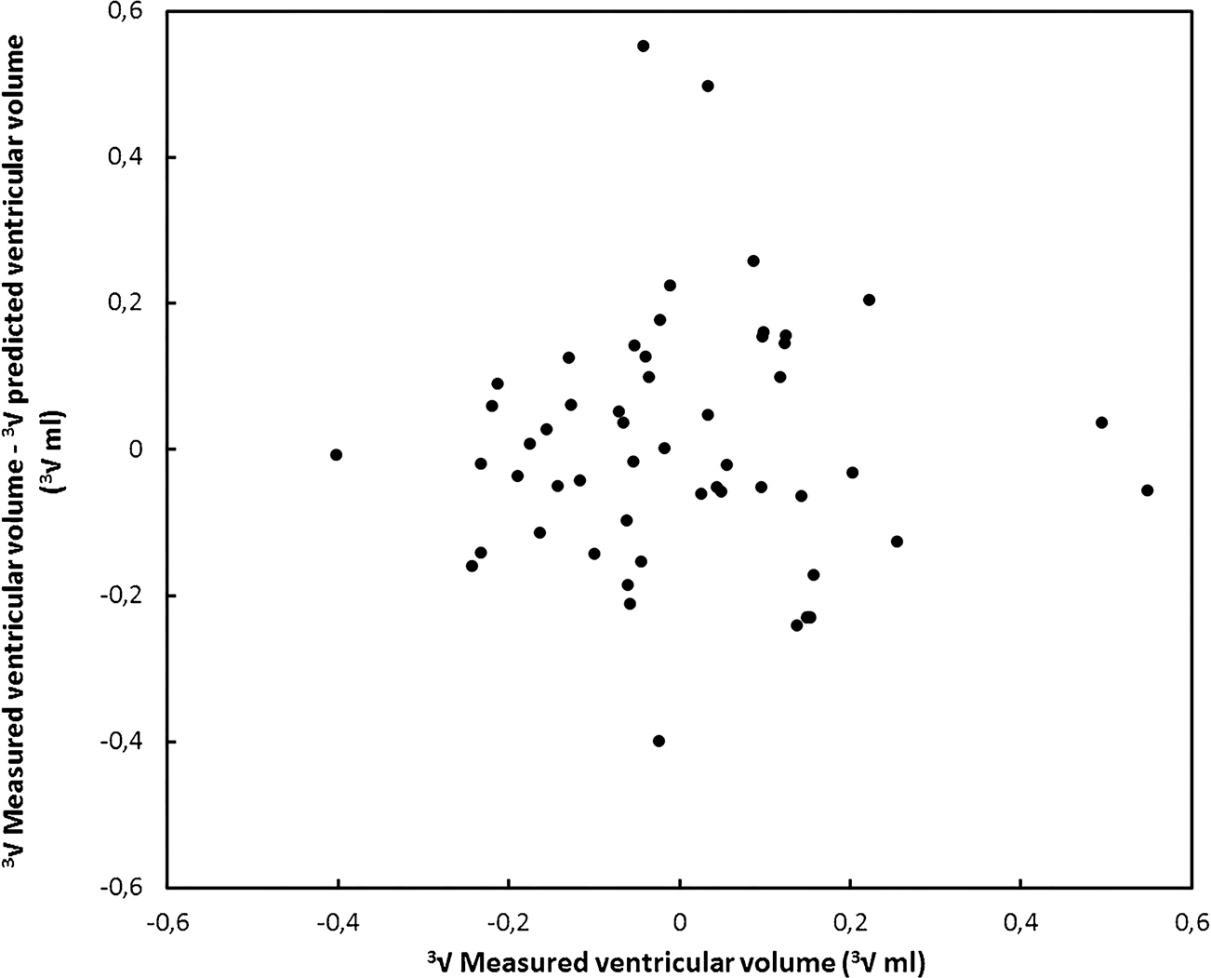
The residual ventricular volume plotted as a function of the ventricular volume in the cube root domain for the model described in this study

We fitted a general linear model to the data using forward variable selection (*P*<0.05) to obtain a model that can be used to predict the ventricular volume using 2-D cranial US measurements. For further analysis we chose to use a transformation with the cube root rather than a log transformation because this better explains the relationship between the physical quantities. That is, the model predicts volumes that scale as the third power of the linear measurements. Forward variable selection showed that the model with anterior horn width and thalamo-occipital distance had the highest adjusted R^2^ and provided a fit with the most symmetrical distribution of residuals, resulting in a model that can be used to predict ventricular volume (∛ventricular volume [ml] = 1.096+0.094 × anterior horn width + 0.020 × thalamo-occipital distance [mm] with R^2^ = 0.831). More details are given in Table 3. The residuals of our model are shown in Fig. 4.

**Table 3:** Parameter values of the univariate general linear model with the parameters anterior horn width and thalamo-occipital distance and the cube root of the ventricular volume as the independent variables

Parameter	Parameter value	*P*	95% confidence interval	
			Lower bound	Upper bound
**a**	1.096	<0.001	.969	1.224
**b**	0.094	<0.001	.072	.116
**c**	0.020	<0.001	.013	.028

R^2^ = 0.831 (Adjusted R^2^ = 0.825)

∛ventricular volume (ml) = a + b × anterior horn width (mm) + c × thalamo-occipital distance (mm)

**Fig. 5:**
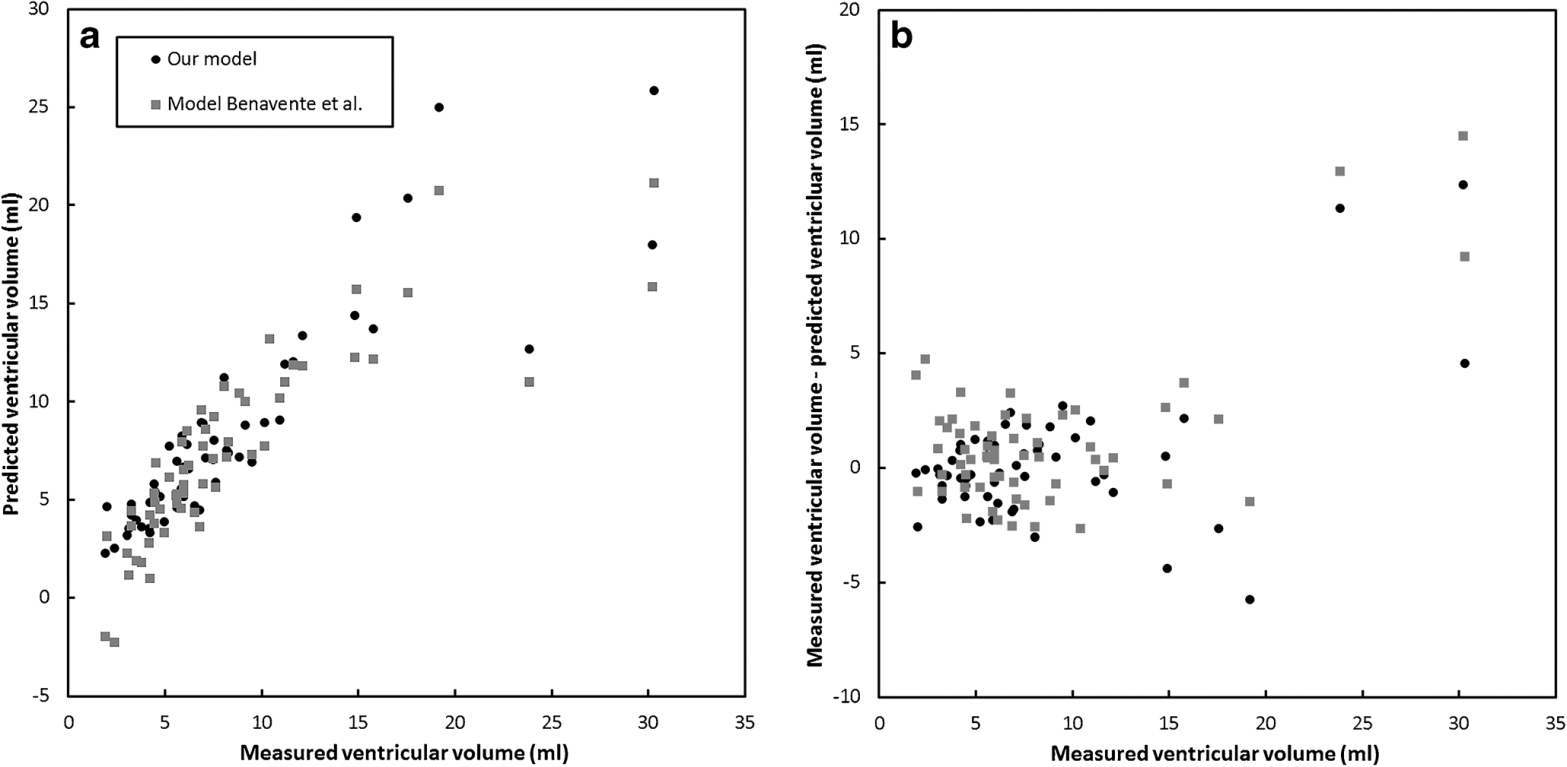
Comparison of the model described in this study and the model obtained by Benavente-Fernandez et al. [13]. **a** With the predicted ventricular volume plotted as a function of the measured ventricular volume. **b** With the residual ventricular volume plotted as a function of the ventricular volume

The model as described here was validated against the model of Benavente-Fernandez et al. [13] (ventricular volume [ml] = −11.02+0.668 × ventricular index [mm] + 0.817 × anterior horn width [mm] + 0.256 × thalamo-occipital distance [mm]) [13]. Figure 5 shows the predicted ventricular volume as a function of the measured ventricular volume for both models and the residual error as a function of the measured ventricular volume for both models. The figure shows that larger discrepancies can be observed for ventricular volume greater than 20 ml, whereas smaller discrepancies of up to 5 ml were observed for ventricular volumes smaller than 20 ml.

## Discussion

In this study, we found that 2-D cranial US ventricular measurements of infants with germinal matrix intraventricular haemorrhage were significantly associated with the volume of the ventricles as determined with MR images in a wide range of ventricular volumes. The proposed model provides us with an estimate of the ventricular volume that can be obtained by performing only a few linear measurements on US images, which enables bedside estimation of ventricular volume. This highlights the utility of cranial US in the diagnosis and monitoring of preterm infants with post-haemorrhagic ventricular dilatation, and should encourage neonatologists to perform linear cranial US measurements of the cerebral ventricles. Figure 5 shows us that larger deviations from the model seem to appear for volumes over 20 ml. Although more data are needed to confirm this hypothesis, it seems that the model struggles to predict the larger volumes accurately. This might be explained by the fact that the shape of the ventricles changes considerably for larger volumes, so the estimation of the volume based on two local linear measurements might be more likely to fail. We argue that this does not necessarily hamper the use of the model in clinical practice. Infants with ventricular volumes higher than 20 ml undergo MRI regardless of the exact results of the US examination. Especially for smaller ventricular volumes, the model might aid in the discrimination between healthy infants and those with mild cases of germinal matrix intraventricular haemorrhage.

Other relevant work on the prediction of ventricular volume using US has been performed by Benavente-Fernandez et al. [13]. They created a model to predict the ventricular volume as measured by 3-D cranial US using linear 2-D cranial US measurements for infants with post-haemorrhagic ventricular dilatation. There were two major differences between this model (ventricular volume = a + b × ventricular index + c × anterior horn width + d × thalamo-occipital distance) and our model (∛ventricular volume = a + b × anterior horn width + c × thalamo-occipital distance) that might explain the observed discrepancies. First, the Benavente-Fernandez model included the ventricular index, anterior horn width and thalamo-occipital distance, whereas we only included the anterior horn width and thalamo-occipital distance. Second, their model did not perform a cube root transformation of the ventricular volume (∛ventricular volume). Other differences might be explained by the fact that 3rd and 4th ventricles were not included in the 3-D volume measurements by Benavente-Fernandez et al. [13], whereas they were included in our segmentations. The 3rd ventricle is hard to visualize on US imaging and, hence, few reference values are known in the literature. Moreover, the relative overestimation of ventricular volume on MRI as compared to 3-D cranial US has also been reported by Kishimoto et al. [14], which might be attributed to the fact that the posterior horns are difficult to visualize.

Limitations of this retrospective study include errors in the measurement of the variables apart from the variance in the data owing to the anatomical variation among patients and the intraobserver variability. For example, errors in the measurement of the ventricular index, anterior horn width and thalamo-occipital distance with 2-D cranial US might have occurred because of the absence of contrast between the borders of the ventricles and the rest of the surrounding brain tissue, especially for the occipital horns. Errors in the measurement of the ventricular volume on MRI might be caused by susceptibility artifacts or partial volume effects. Finally, errors in the model parameters could have been introduced by differences between the actual volume of the ventricles at the time of the MRI and 2-D cranial US acquisition; to minimize this difference, we chose a maximum of 3 days between the MRI and 2-D cranial US acquisition.

The reliability of our model is affected by the variance of linear 2-D cranial US measurements and because few data points are available for ventricles with a large volume. The fact that linear measurements with large variance are used, and that the distribution of several of the measured parameters is asymmetrical, could impact the prediction. This could also explain the fact that larger discrepancies between our model and the Benavente-Fernandez model were observed for larger volumes.

Although this study aimed to improve ventricular volume determination in clinical practice, the relation between ventricular volume and intracranial pressure — which might be the actual cause of damage — remains unclear, and additional investigations performed simultaneously, such as near infrared spectroscopy (NIRS), amplitude integrated electroencephalogram (EEG) and Doppler measurements of cerebral blood flow might provide additional important information [15, 16].

## Conclusion

With the use of 2-D cranial US an approximation of the ventricular volume can be provided, which might be helpful for better management of post-haemorrhagic ventricular dilatation. Validation of our model in a larger study group including different time points and ventricular sizes is warranted.
